# Causal association between lipid-lowering drugs and cancers: A drug target Mendelian randomization study

**DOI:** 10.1097/MD.0000000000038010

**Published:** 2024-05-03

**Authors:** Wenjing Ding, Liangliang Chen, Jianguo Xia, Bei Pei, Biao Song, Xuejun Li

**Affiliations:** a The Second Clinical Medical School, Anhui University of Chinese Medicine, Hefei, Anhui, China; b Department of Gastroenterology, The Second Affiliated Hospital of Anhui University of Chinese Medicine, Hefei, Anhui, China.

**Keywords:** cancers, drug target Mendelian randomization, HMGCR, lipid-lowering drugs, PCSK9

## Abstract

Accumulating evidences have indicated that lipid-lowering drugs have effect for the treatment of cancers. However, causal associations between lipid-lowering drugs and the risk of cancers are still unclear. In our study, we utilized single nucleotide polymorphisms of proprotein convertase subtilis kexin 9 (PCSK9) inhibitors and 3-hydroxy-3-methylglutaryl-assisted enzyme A reductase (HMGCR) inhibitors and performed a drug target Mendelian randomization to explore the causal association between lipid-lowering drugs and the risk of cancers. Five regression methods were carried out, including inverse variance weighted (IVW) method, MR Egger, weighted median, simple mode and weighted mode methods, of which IVW method was considered as the main analysis. Our outcome dataset contained the risk of breast cancer (BC), colorectal cancer, endometrial cancer, gastric cancer (GC), hepatocellular carcinoma (HCC), lung cancer, esophageal cancer, prostate cancer (PC), and skin cancer (SC). Our results demonstrated that PCSK9 inhibitors were significant associated with a decreased effect of GC [IVW: OR = 0.482, 95% CI: 0.264–0.879, *P = .*017]. Besides, genetic inhibitions of HMGCR were significant correlated with an increased effect of BC [IVW: OR = 1.421, 95% CI: 1.056–1.911, *P* = .020], PC [IVW: OR = 1.617, 95% CI: 1.234–2.120, *P* = .0005] and SC [IVW: OR = 1.266, 95% CI: 1.022–1.569, *P* = .031]. For GC [IVW: OR = 0.559, 95% CI: 0.382–0.820, *P* = .0029] and HCC [IVW: OR = 0.241, 95% CI: 0.085–0.686, *P* = .0077], HMGCR inhibitors had a protective risk. Our method suggested that PCSK9 inhibitors were significant associated with a protective effect of GC. Genetic inhibitions of HMGCR were significant correlated with an increased effect of BC, PC and SC. Meanwhile, HMGCR inhibitors had a protective risk of GC and HCC. Subsequent studies still needed to assess potential effects between lipid-lowering drugs and the risk of cancers with clinical trials.

## 1. Introduction

Cancer is one of the most prevalent diseases all over the world and it accounts for around 10 million deaths in 2020 as reported in.^[1]^ Hence, many researchers focus on the treatment of cancers to relieve pain of patients. The generation of tumor has many reasons, for instance, inheritance, stress, daily diet, and environment.^[2]^ As aforementioned in epidemiological studies,^[3]^ the cholesterol is related to the development of cancer, but causal associations between cholesterol levels and tumorigenesis were still unclear and controversial.^[4,5]^ In a nutshell, it is vital for us to explore causal associations between lipid-lowering drugs, which can regulate low-density lipoprotein cholesterol (LDL-C), and cancers.

Proprotein convertase subtilis kexin 9 (PCSK9) inhibitors, for instance, alirocumab and evolocumab,^[6]^ are now considered to protect against hypercholesterolemia and coronary heart disease based on lipid-lowering therapeutic.^[7]^ Although reduced effects of PCSK9 inhibitors in coronary heart disease have been understood clearly,^[8]^ causal associations between PCSK9 inhibitors and cancers are still unclear. A recent study have reported that evolocumab and alirocumab have no influence on cancer risk based on several large sample-size clinical trials.^[9]^ However, some previous preclinical trials have suggested that PCSK9 inhibitors were involved in carcinogenesis, for example, hepatocellular carcinoma,^[10]^ lung cancer,^[11]^ breast cancer (BC)^[12]^ and so on. 3-Hydroxy-3-methylglutaryl-assisted enzyme A reductase (HMGCR) inhibitors (statins) are considered as lipid-lowering drugs, which have been proven effective for cardiovascular diseases.^[13]^ According to pharmacokinetics studies, statins can cause cytostatic and cytotoxic results of cancer cells to induce apoptosis^[14]^ and arrest both G1 and S phase in vitro studies.^[15,16]^ However, accurate principle of HMGCR inhibitors is still equivocal. Above studies have demonstrated that both PCSK9 inhibitors and HMGCR inhibitors may have influence on the tumorigenesis through a various of methods. Nevertheless, causal relationships between PCSK9/HMGCR inhibitors and cancers need to be explored subsequently.

The drug target Mendelian randomization (MR)^[17]^ utilizes single nucleotide polymorphisms (SNPs) as instrumental variables (IVs) from medications to explore causal associations between exposure dataset (e.g., lipid-lowering drugs) and outcome dataset based on genome-wide association study (GWAS) summary data. In our drug target MR study, genetic variants encode as drug targets, which were considered as instruments.^[12]^ Genetic variants of PCSK9 and HMGCR were used as genetically proxied PCSK9 and HMGCR inhibitors. This regression method elucidated the underlying effect of long-term lipid-lowering drugs on outcome sources. In our context, lipid-lowering drugs (PCSK9 inhibitors and HMGCR inhibitors) were considered as exposure dataset and 9 selected cancers, including BC, colorectal cancer (CC), endometrial cancer (EC), gastric cancer (GC), hepatocellular carcinoma (HCC), lung cancer (LC), esophageal cancer (OC), prostate cancer (PC) and skin cancer (SC) were considered as outcome dataset.

## 2. Materials and methods

### 2.1. Exposure dataset selection of PCSK9 and HMGCR

In our study, we selected SNPs as IVs in GWAS summary data with 440,546 individuals^[8]^ within ±100 kb window from lipid-lowering drugs as exposure dataset. The selection of SNPs of PCSK9 gene (chromosome 1: 55505221–55530525) and HMGCR gene (chromosome 5: 74632154–74657929) loci via IVs was associated with LDL-C levels. Variants in PSCK9 and HMGCR were utilized as genetic proxies for pharmacological inhibitions of PCSK9 and HMGCR. Meanwhile, we excluded strong linkage disequilibrium based on set threshold *r*^2^ < 0.3. Finally, *F*-statistic values were computed and a high *F* statistical value (*F* > 10) suggested that the weak instrument was unlikely. We obtained 32 significant SNPs of PCSK9 inhibitors and 12 significant SNPs of HMGCR inhibitors.^[8]^

### 2.2. Outcome dataset selection of cancers

In our drug target MR analysis, we employed 9 common cancers as outcome sources. All of these were from GWAS dataset (https://gwas.mrcieu.ac.uk/), including BC with 257,730 individuals, CC with 470,002 individuals, EC with 492,803, GC with 476,116 individuals, HCC with 197,611 individuals, LC with 492,803 individuals, OC with 372,756 individuals, PC with 211,227 individuals and SC with 492,203 individuals, as shown in Table 1. All of dataset employed in our drug target MR study were large-scale public GWAS summary data. Ethical approval and consent to participate were acquired in all original studies.

**Table 1:** The detailed GWAS data information of MR study on causal relationships of lipid-lowering drugs and cancers drugs and cancers.

	Population	Sample size	Number of SNPs	ID
Breast cancer	European	257,730	24,133,589	ebi-a-GCST90018799
Colorectal cancer	European	470,002	24,182,361	ebi-a-GCST90018808
Endometrial cancer	European	240,027	24,135,295	ebi-a-GCST90018838
Gastric cancer	European	476,116	24,188,662	ebi-a-GCST90018849
Hepatocellular carcinoma	East Asian	197,611	8885,115	bbj-a-158
Lung cancer	European	492,803	24,188,684	ebi-a-GCST90018875
Esophageal cancer	European	372,756	8970,465	ieu-b-4960
Prostate cancer	European	211,227	24,119,306	ebi-a-GCST90018905
Skin cancer	European	492,203	24,178,924	ebi-a-GCST90018921

### 2.3. Drug target MR analysis

For our drug target MR study, there are 3 core assumptions to obey. First and foremost, IVs from GWAS summary data should be related to LDL-C, which were utilized to proxy PCSK9 inhibitors (e.g., alirocumab) and HMGCR inhibitors (e.g., statins), respectively. Subsequently, IVs have no associations with confounders. Finally, selected IVs do not have causal associations with outcome cancers via genetically proxied PCSK9 inhibitors and HMGCR inhibitors. Hence, PhenoScanner (http://www.phenoscanner.medschl.cam.ac.uk/) tool was leveraged to remove SNP associated with BC, CC, EC, GC, HCC, LC, OC, PC, and SC directly. After harmonizing drug target IVs with outcome cancers, we utilized 5 MR statistical regression methods for further analysis, including inverse variance weighted (IVW) method,^[18]^ MR Egger,^[19]^ weighted median,^[20]^ simple mode, and weighted mode methods,^[21]^ of which IVW method was deemed as the primary method. Moreover, MR-PRESSO method was employed to exclude outliers for further sensitive analysis. When the drug target MR analysis was finished, heterogeneity and horizontal pleiotropy were both obtained by calculation of Cochrane Q value^[22]^ and MR-Egger test, respectively. Leave-one-out method was employed to remove each SNP in turn to evaluate whether individual SNPs have independent bias that has influence on our drug target MR estimate results. All of our MR statistical analysis were implemented on “TwoSampleMR” (v 0.5.7)^[23]^ and “MR-PRESSO” (v 1.0)^[24]^ packages in R v 4.3.1.

## 3. Results

**Figure 1.**
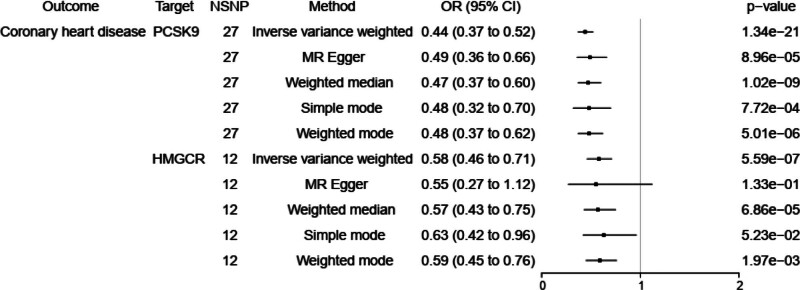
The effect of PCSK9, HMGCR inhibitors on CHD. CI = confidence interval; HMGCR = 3-hydroxy-3-methylglutaryl coenzyme A reductase; MR = Mendelian randomization; NSNP = number of single nucleotide polymorphisms; OR = odds ratio; PCSK9 = proprotein convertase subtilisin/kexin 9.

According to drug target MR results in,^[8]^ we chose coronary heart disease (CHD) with 141,217 individuals as positive control analysis. Because exposure dataset of PCSK9 inhibitors and HMGCR inhibitors were both from,^[8]^ it is reasonable for us to leverage MR estimates of aforementioned study between PCSK9/HMGCR inhibitor and CHD to support our drug target MR analysis as shown in Figure 1.

### 3.1. Causal association between PCSK9 inhibitors and cancers

**Figure 2.**
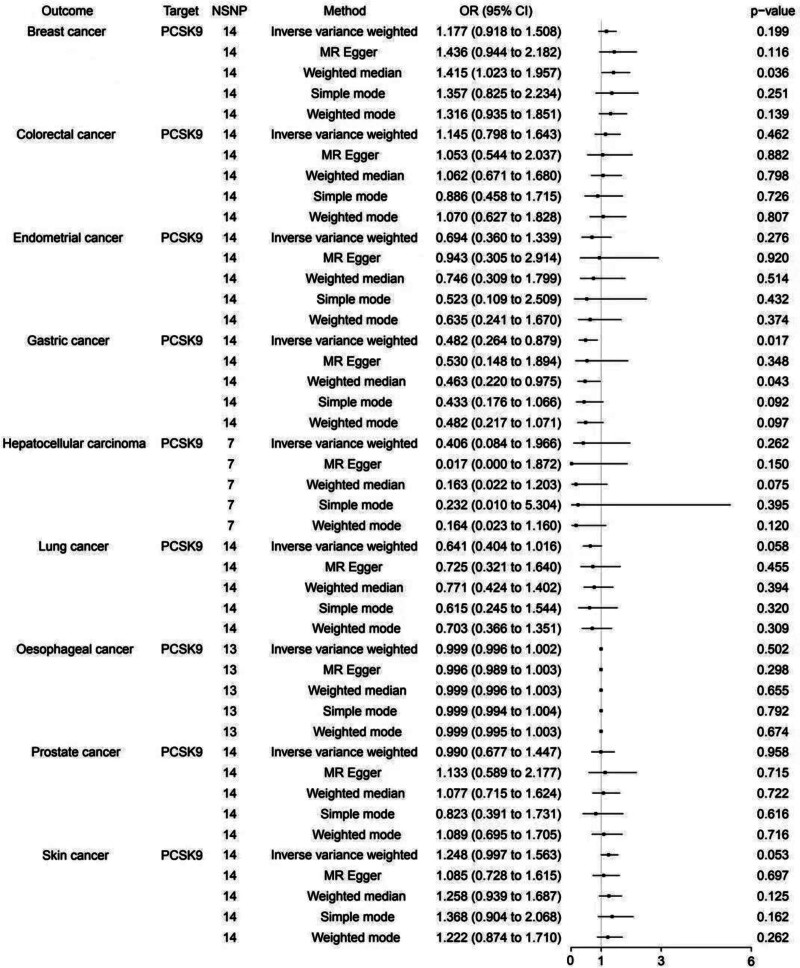
The effect of PCSK9 inhibitors on 9 cancers. CI = confidence interval; MR = Mendelian randomization; NSNP = number of single nucleotide polymorphisms; OR = odds ratio; PCSK9 = proprotein convertase subtilisin/kexin 9.

As shown in Figure 2, genetically proxied PCSK9 inhibitors were statistically correlated with a reduced effect of GC [IVW: OR = 0.482, 95% CI: 0.264–0.879, *P* = .017; MR Egger: OR = 0.530, 95% CI: 0.148–1.894, *P* = .348; weighted median: OR = 0.463, 95% CI: 0.220–0.975, *P* *= .*043; simple mode: OR = 0.433, 95% CI: 0.176–1.066, *P* = .092; weighted mode: OR = 0.482, 95% CI: 0.217–1.071, *P* = .097]. On the other hand, PCSK9 inhibitors were found to have no causal associations with BC [IVW: OR = 1.177, 95% CI: 0.918–1.508, *P* = .199; MR Egger: OR = 1.436, 95% CI: 0.944–2.182, P = .116; weighted median: OR = 1.415, 95% CI: 1.023–1.957, *P* = .036; simple mode: OR = 1.357, 95% CI: 0.825–2.234, *P* = .251; weighted mode: OR = 1.316, 95% CI: 0.935–1.851, *P* = .139], CC [IVW: OR = 1.145, 95% CI: 0.798–1.643, *P* = .462; MR Egger: OR = 1.053, 95% CI: 0.544–2.037, *P* = .882; weighted median: OR = 1.062, 95% CI: 0.671–1.680, *P* = .798; simple mode: OR = 0.886, 95% CI: 0.458–1.715, *P* = .726; weighted mode: OR = 1.070, 95% CI: 0.627–1.828, *P* = .807], EC [IVW: OR = 0.694, 95% CI: 0.360–1.339, *P* = .276; MR Egger: OR = 0.943, 95% CI: 0.305–2.914, *P* = .920; weighted median: OR = 0.746, 95% CI: 0.309–1.799, *P* = .514; simple mode: OR = 0.523, 95% CI: 0.109–2.509, *P* = .432; weighted mode: OR = 0.635, 95% CI: 0.241–1.670, *P* = .374], HCC [IVW: OR = 0.406, 95% CI: 0.084–1.966, *P* = .262; MR Egger: OR = 0.017, 95% CI: 0.000–1.872, *P* = .150; weighted median: OR = 0.163, 95% CI: 0.022–1.203, *P* = .075; simple mode: OR = 0.232, 95% CI: 0.010–5.304, *P* = .395; weighted mode: OR = 0.164, 95% CI: 0.023–1.160, *P* = .120], LC [IVW: OR = 0.641, 95% CI: 0.404–1.016, *P* = .058; MR Egger: OR = 0.725, 95% CI: 0.321–1.640, *P* = .455; weighted median: OR = 0.771, 95% CI: 0.424–1.402, *P* = .394; simple mode: OR = 0.615, 95% CI: 0.245–1.544, *P* = .320; weighted mode: OR = 0.703, 95% CI: 0.366–1.351, *P* = .309], OC [IVW: OR = 0.999, 95% CI: 0.996–1.002, *P* = .502; MR Egger: OR = 0.996, 95% CI: 0.989–1.003, *P* = .298; weighted median: OR = 0.999, 95% CI: 0.996–1.003, *P* = .655; Simple mode: OR = 0.999, 95% CI: 0.994–1.004, *P* = .792; Weighted mode: OR = 0.999, 95% CI: 0.995–1.003, *P* = .674], PC [IVW: OR = 0.990, 95% CI: 0.677–1.447, *P* = .958; MR Egger: OR = 1.133, 95% CI: 0.589–2.177, *P* = .715; weighted median: OR = 1.077, 95% CI: 0.715–1.624, *P* = .722; simple mode: OR = 0.823, 95% CI: 0.391–1.731, *P* = .616; weighted mode: OR = 0.999, 95% CI: 0.995–1.003, *P* = .674], SC [IVW: OR = 1.248, 95% CI: 0.997–1.563, *P* = .053; MR Egger: OR = 1.085, 95% CI: 0.728–1.615, *P = .*697; weighted median: OR = 1.258, 95% CI: 0.939–1.687, *P* = .125; simple mode: OR = 1.368, 95% CI: 0.904–2.068, *P* = .162; weighted mode: OR = 1.222, 95% CI: 0.874–1.710, *P* = .262] based on the primary IVW regression method.

### 3.2. Causal association between HMGCR inhibitor and cancers

**Figure 3.**
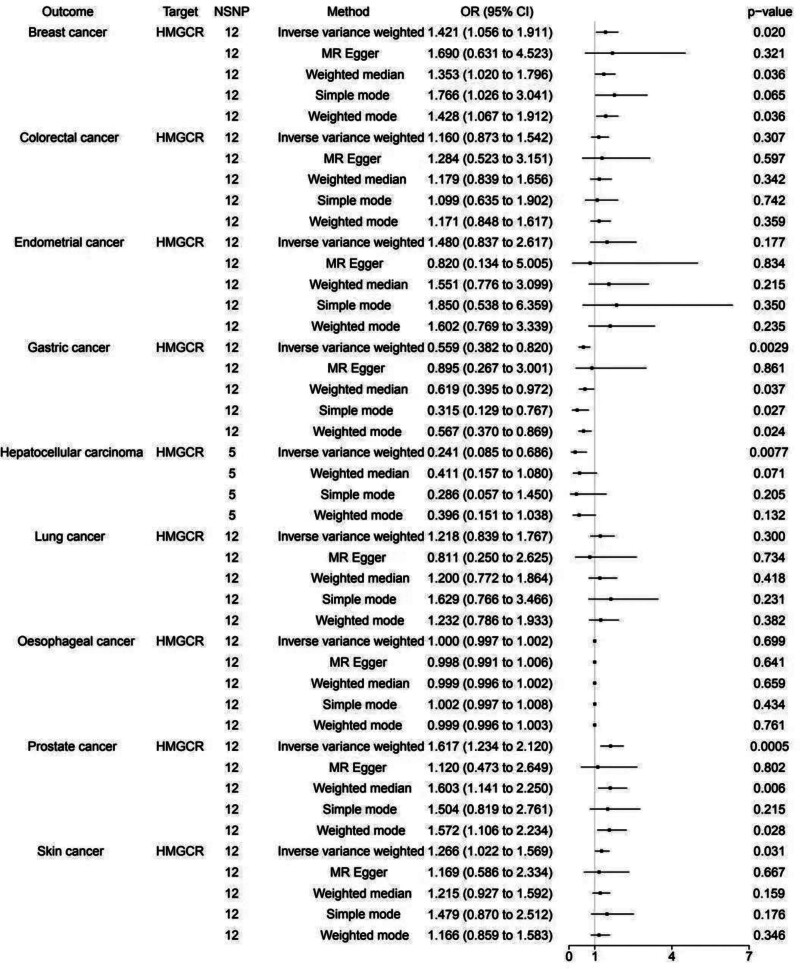
The effect of HMGCR inhibitors on 9 cancers. CI = confidence interval; HMGCR = 3-hydroxy-3-methylglutaryl coenzyme A reductase; MR, Mendelian randomization; NSNP = number of single nucleotide polymorphisms; OR = odds ratio.

The drug target MR statistical analysis revealed causal associations between genetically proxied HMGCR inhibitors and 9 cancers, which were outlined in Figure 3. As shown in the main IVW regression method, HMGCR inhibitors had a positive effect for BC [IVW: OR = 1.421, 95% CI: 1.056–1.911, *P* = .020; MR Egger: OR = 1.690, 95% CI: 0.631–4.523, *P* = .321; weighted median: OR = 1.353, 95% CI: 1.020–1.796, *P* = .036; simple mode: OR = 1.766, 95% CI: 1.026–3.041, *P* = .065; weighted mode: OR = 1.428, 95% CI: 1.067–1.912, *P* = .036], PC [IVW: OR = 1.617, 95% CI: 1.234–2.120, *P* = .0005; MR Egger: OR = 1.120, 95% CI: 0.473–2.649, *P* = .802; weighted median: OR = 1.603, 95% CI: 1.141–2.250, *P* = .006; simple mode: OR = 1.504, 95% CI: 0.819–2.761, *P* = .215; weighted mode: OR = 1.572, 95% CI: 1.106–2.234, *P* = .028] as well as SC [IVW: OR = 1.266, 95% CI: 1.022–1.569, *P = .*031; MR Egger: OR = 1.169, 95% CI: 0.586–2.334, *P = .*667; weighted median: OR = 1.215, 95% CI: 0.927–1.592, *P = .*159; simple mode: OR = 1.479, 95% CI: 0.870–2.512, *P* = .176; Weighted mode: OR = 1.166, 95% CI: 0.859–1.583, *P* = .346]. Meanwhile, genetically predicted inhibitions of HMGCR had a protective risk of GC [IVW: OR = 0.559, 95% CI: 0.382-0.820, *P = .*0029; MR Egger: OR = 0.895, 95% CI: 0.267–3.001, *P* = .861; weighted median: OR = 0.619, 95% CI: 0.395–0.972, *P* = .037; simple mode: OR = 0.315, 95% CI: 0.129–0.767, *P* = .027; Weighted mode: OR = 0.567, 95% CI: 0.370–0.869, *P* = .024] and HCC [IVW: OR = 0.241, 95% CI: 0.085–0.686, *P* = .0077; weighted median: OR = 0.411, 95% CI: 0.157–1.080, *P* = .071; Simple mode: OR = 0.286, 95% CI: 0.057–1.450, *P* = .205; weighted mode: OR = 0.396, 95% CI: 0.151–1.038, *P* = .132]. However, HMGCR inhibitors showed no drug target MR association with CC [IVW: OR = 1.160, 95% CI: 0.873–1.542, *P* = .307; MR Egger: OR = 1.284, 95% CI: 0.523–3.151, *P* = .597; weighted median: OR = 1.179, 95% CI: 0.839–1.656, *P* = .342; simple mode: OR = 1.099, 95% CI: 0.635–1.902, *P* = .742; weighted mode: OR = 1.171, 95% CI: 0.848–1.617, *P* = .359], EC [IVW: OR = 1.480, 95% CI: 0.837–2.617, *P* = .177; MR Egger: OR = 0.820, 95% CI: 0.134–5.005, *P* = .834; weighted median: OR = 1.551, 95% CI: 0.776–3.099, *P = .*215; simple mode: OR = 1.850, 95% CI: 0.538–6.359, *P* = .350; weighted mode: OR = 1.602, 95% CI: 0.769–3.339, *P* = .235], LC [IVW: OR = 1.218, 95% CI: 0.839–1.767, *P* = .300; MR Egger: OR = 0.811, 95% CI: 0.250–2.625, *P* = .734; weighted median: OR = 1.200, 95% CI: 0.772–1.864, *P* = .418; simple mode: OR = 1.629, 95% CI: 0.766–3.466, *P* = .231; weighted mode: OR = 1.232, 95% CI: 0.786–1.933, *P* = .382] and OC [IVW: OR = 1.000, 95% CI: 0.997–1.002, *P = .*699; MR Egger: OR = 0.998, 95% CI: 0.991–1.006, *P* = .641; weighted median: OR = 0.999, 95% CI: 0.996–1.002, *P = .*659; simple mode: OR = 1.002, 95% CI: 0.997–1.008, *P = .*434; weighted mode: OR = 0.999, 95% CI: 0.996–1.003, *P = .*761].

### 3.3. Robustness analysis

Cochran Q and MR-Egger test methods were employed to evaluate heterogeneity and horizontal pleiotropy. As shown in Table 2, robustness analysis results revealed that there were no evidence of statistically significant heterogeneity and horizontal pleiotropy for all *P* > .05. Meanwhile, leave-one-out analysis was performed and it indicated that excluding any SNPs from selected cancers had no impact on our drug target MR statistical analysis, as shown in Figure S1, Supplemental Digital Content, http://links.lww.com/MD/M316 leaveoneout PCSK9 and Figure S2, Supplemental Digital Content, http://links.lww.com/MD/M317 leaveoneout HMGCR.

**Table 2:** IVW heterogeneity test and MR-Egger pleiotripy test between lipid-lowering drugs and cancers.

Exposure	Outcome	IVW heterogeneity test		MR-Egger pleiotropy test	
		Q	*P* value	Intercept	*P* value
PCSK9 inhibitor	Breast cancer	8.56	.81	−0.008	.27
HMGCR inhibitor		18.92	.06	−0.008	.72
PCSK9 inhibitor	Colorectal cancer	8.17	.83	3.14E−03	.77
HMGCR inhibitor		2.27	.1	−0.005	.82
PCSK9 inhibitor	Endometrial cancer	13.68	.4	−0.01	.52
HMGCR inhibitor		5.97	.88	0.03	.52
PCSK9 inhibitor	Gastric cancer	6.93	.91	−0.003	.87
HMGCR inhibitor		0.52	.42	−0.02	.44
PCSK9 inhibitor	Hepatocellular carcinoma	5.52	.48	8.26E−02	.22
HMGCR inhibitor		6.62	.16	−1.20E−01	.13
PCSK9 inhibitor	Lung cancer	12.33	.5	−0.005	.72
HMGCR inhibitor		8.05	.71	0.02	.49
PCSK9 inhibitor	Oesophageal cancer	9.04	.7	8.93E−05	.39
HMGCR inhibitor		8.4	.68	6.49E−05	.71
PCSK9 inhibitor	Prostate cancer	20.73	.08	−0.006	.62
HMGCR inhibitor		6.84	.81	0.02	.4
PCSK9 inhibitor	Skin cancer	7.23	.89	0.006	.42
HMGCR inhibitor		4.97	.93	0.004	.82

* HMGCR = 3-Hydroxy-3-methylglutaryl-assisted enzyme A reductase, IVW = inverse variance weighted, PCSK9 = proprotein convertase subtilis kexin 9.

## 4. Discussion

For drug target MR studies, our research is the first study to assess causal associations between lipid-lowering drugs and cancers. Genetic variants in PSCK9 and HMGCR were utilized as a proxy for pharmacological PCSK9 and HMGCR inhibitors. Our findings suggested that PCSK9 inhibitors were significant correlated to a decreased effect of GC [IVW: OR = 0.482, 95% CI: 0.264–0.879, *P* = .017]. On the other hand, our study found out that genetic inhibitions of HMGCR were significant correlated with an increased effect of BC [IVW: OR = 1.421, 95% CI: 1.056–1.911, *P* = .020], PC [IVW: OR = 1.617, 95% CI: 1.234–2.120, *P* = .0005] and SC [IVW: OR = 1.266, 95% CI: 1.022–1.569, *P* = .031]. For GC [IVW: OR = 0.559, 95% CI: 0.382–0.820, *P* = .0029] and HCC [IVW: OR = 0.241, 95% CI: 0.085–0.686, *P* = .0077], HMGCR inhibitors had a protective risk.

It is noteworthy that PCSK9 inhibitors have a risk-decreasing effect on GC in our drug target MR study. GC is one of the most common globally important disease,^[25]^ which can be related to high LDL-C.^[26]^ However, a previous study have demonstrated that low LDL-C can also cause the risk of GC^[27]^ or no associations.^[28]^ A quantitative study has manifested that PCSK9 as a potential biomarker was 4-fold higher in the neoplastic gastric epithelial cells.^[29]^ Given that the underlying mechanism by which PCSK9 was involved in gastric cancer is still uncertain, a vitro-vivo study has indicated that PCSK9 promoted invasion and suppressed apoptosis of GC. Meanwhile, it can also cause the promotion of GC metastasis and suppress apoptosis based on facilitating MAPK signaling pathway, which is correlated with proliferation, differentiation and apoptosis of cancer cells.^[30]^ Besides, our studies found that HMGCR inhibitors were associated with raised BC, PC, and SC risk, while GC and HCC had opposite associations. In line with this, an experimental study has shown that 5 µM lovastatin (HMGCR inhibitors) can arrest proteasome of BC cells, which BC cells failed to enter the G1 phase.^[31]^ HMGCR enzyme, which was considered as a biomarker, has been demonstrated that intake of short-term atorvastatin with high dose can reduce proliferation of HMGCR-positive BC.^[32]^ Nevertheless, a recent study has reported that HMGCR inhibitors suppressed phosphorylation and activity of protein kinase B in PC cells and also arrested proliferation, migration, invasion and apoptosis of PC cells.^[33]^ However, less literature investigated causal relationships between HMGCR inhibitors and SC except an updated meta-analysis^[34]^ with risk-decreasing associations. The discrepancy may be due to shortcomings of both MR analysis and meta-analysis. In,^[35]^ this study found out that overexpression of HMGCR promoted the proliferation and migration of GC cells, which activated Hedgehog/Gli1 signaling way and facilitated the manifestation of Gli1 target genes in GC cells. For HCC, a recent study found out that HMGCR inhibitions were able to rewire lipid metabolism of HCC cells, damaging their proliferation, migration and energetic metabolism based on a chicken choriorallantoic membrane model.^[36]^

Moreover, our drug target MR study failed to reach nominal significance of associations between PCSK9 inhibitors and BC, CC, EC, HCC, LC, OC, PC, and SC. Excitingly, a previous MR study has found a relationship between increased LDL-C variants mimicking inhibition of PCSK9 and BC.^[12]^ The strong causal link between genetically proxied PCSK9 inhibition and a lower risk of total and early-onset PC was evaluated based on a drug target MR study.^[37]^ The conflicts of drug target MR results may be owing to different selection of GWAS dataset. In a mouse model of experimental research, the inhibition of PCSK9 suppressed growth of CC by anti-PCSK9 (L-IFPTA+) vaccine.^[38]^ To date, there are no work to investigate between PCSK9 inhibitors and EC. Only one study incidentally mentioned that western blot analysis of OC cell line lysates identified variable expression of PCSK9 isoforms in comparison with HeLa cell, of which its line cell type is EC.^[39]^ Additionally, a human study has documented that reduced PCSK9 promoted the growth of HCC, and PCSK9 inhibitors with antibodies or targeted pharmaceuticals may be protective against the metabolism of HCC.^[40]^ Findings from a study in^[41]^ reported that PCSK9 siRNA provides an antitumor potency through mitochondrial pathway in A549 human LC cells. Moreover, evidences from^[42]^ have shown that patients with high serum anti-PCSK9 antibody levels were demonstrated to undergo a favorable prognosis in the case of OC. Conversely, our study did not observe causal associations between HMGCR inhibitors and CC, EC, LC and OC. It has been reported that the overall risk of developing CC is related to the expression of HMGCR with a lack of exon 13.^[43]^ A previous experimental study has acknowledged that simvastatin, one of inhibition of HMGCR activity, had essential antiproliferative and anti-metastatic effects in EC cells via modulation of both the MAPK and AKT/mTOR pathways, and it may be potential drug for the treatment of EC.^[44]^ It has been shown that HMGCR inhibitors had weak effects, which tumor cells failed to express the organic anion-transporting polypeptide OATP1B1 transporter including LC, PC, and SC.^[45]^ Previous randomized controlled trials (RCTs) revealed that statins (HMGCR inhibitors) did not increase overall cancer incidence or cancer mortality,^[46]^ which can prevent BC, CC, PC, and SC effectively.^[47]^ Besides, a high-quality RCT demonstrated that statins had no effect on LC and PC. Meanwhile, it had a protective effect on GC and increased the incidence of SC.^[48]^ A two-sample MR analysis has proven a risk-increasing relationship between lipid-lowering drugs targeting (statins) and HCC,^[49]^ while genetically proxied HMGCR inhibitors were significantly risk-decreasing related to BC.^[50]^ Further RCTs are needed to demonstrate separate roles of HMGCR inhibitors (statins) in overall cancers.

Moreover, our study still had some limitations. First of all, our drug target MR study was theoretical method without the demonstration of clinical trials to assess causal link between lipid-lowering drugs and cancers. Subsequently, most population of our investigative dataset were from European due to limitations of GWAS summary data resources. It may lead to bias estimates and reduce the credibility of the data. Finally, drug target MR estimates can only explore long-term exposure. For short-term treatment effects of drugs, our analysis was inaccurate.

## 5. Conclusion

Taken together, our drug target MR analysis method reported that PCSK9 inhibitors were significant associated with a decreased effect of GC. Besides, it indicated that genetic inhibitions of HMGCR were significant correlated with an increased effect of BC, PC, and SC. For GC and HCC, HMGCR inhibitors had a protective risk.

## Acknowledgments

We would like to thank the supports from the Second Affiliated Hospital of Anhui University of Chinese Medicine and the contributions from the participants in our study and investigators of the database (https://gwas.mrcieu.ac.uk/) used in this study.

## Author contributions

**Conceptualization:** Wenjing Ding, Liangliang Chen, Jianguo Xia, Bei Pei, Xuejun Li.

**Funding acquisition:** Xuejun Li.

**Investigation:** Wenjing Ding.

**Methodology:** Wenjing Ding, Bei Pei, Biao Song, Xuejun Li.

**Visualization:** Wenjing Ding.

**Writing – original draft:** Wenjing Ding.

**Writing – review & editing:** Liangliang Chen, Jianguo Xia, Bei Pei, Biao Song, Xuejun Li.

## Supplementary Material




