# Supplementary figures and images for: Causal association between lipid-lowering drugs and cancers: A drug target Mendelian randomization study
Source: Medicine (Baltimore). 2024 May 3;103(18):e38010. doi: 10.1097/MD.0000000000038010 (PMC11062692; doi:10.1097/MD.0000000000038010)

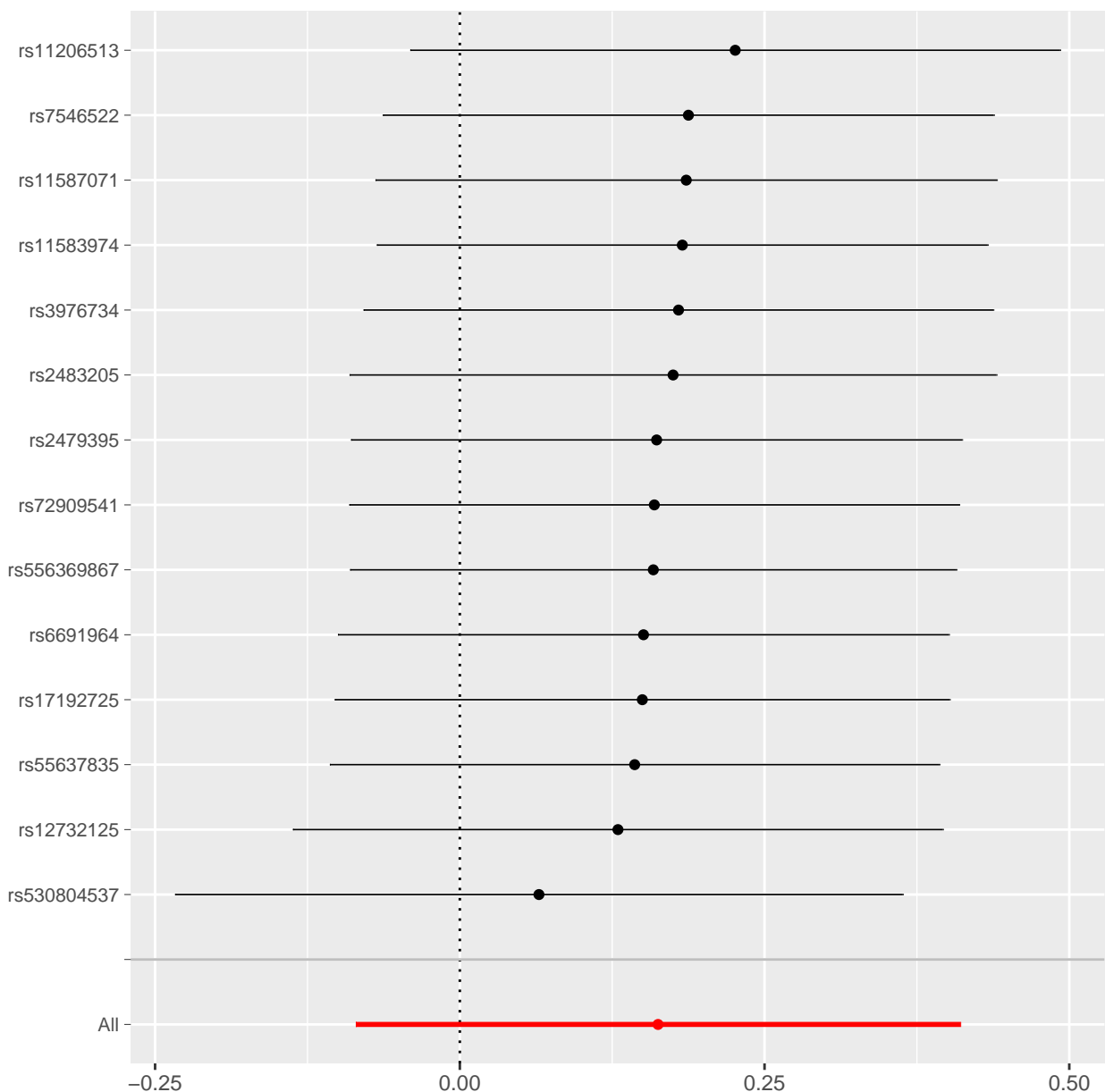

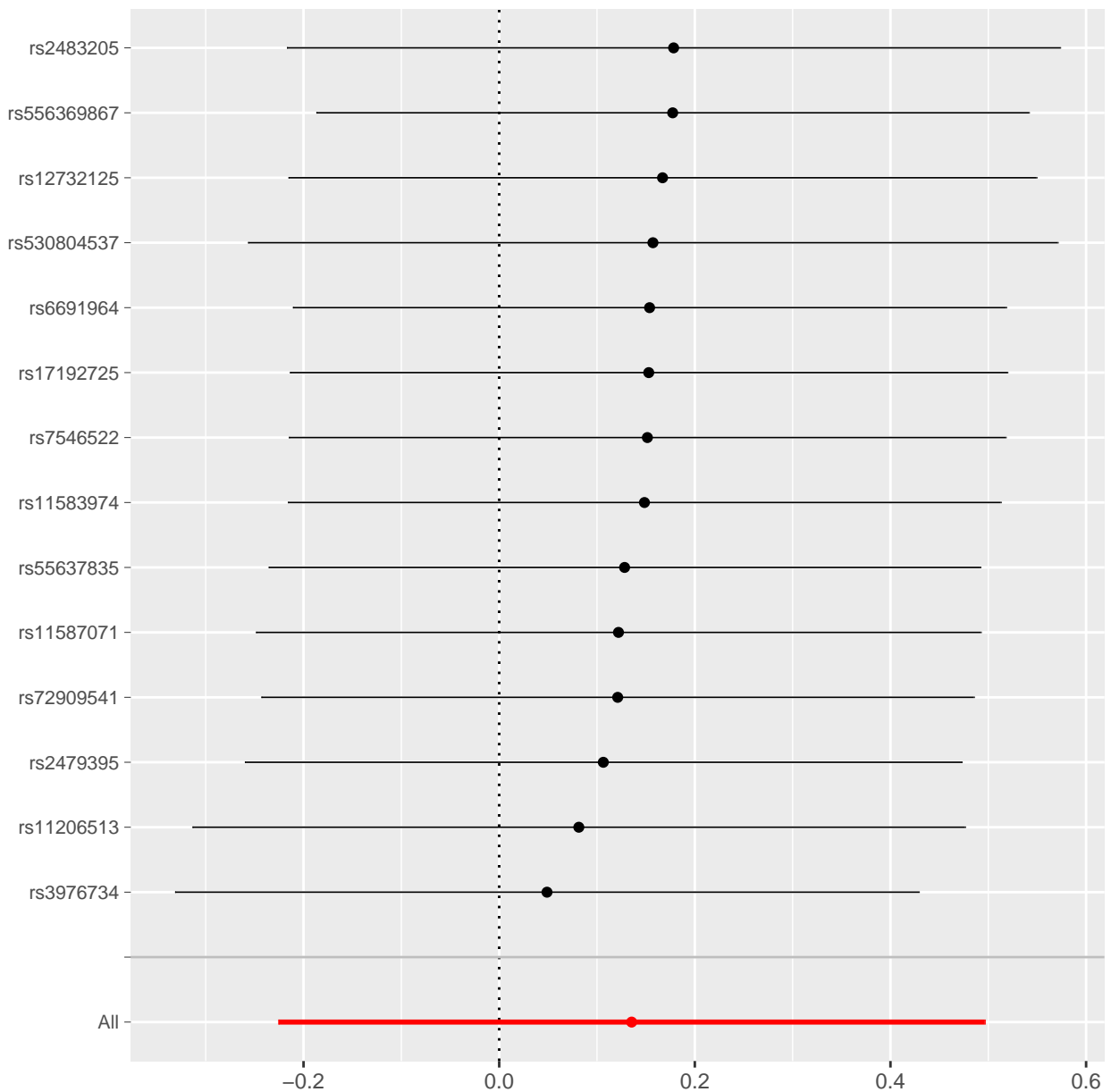

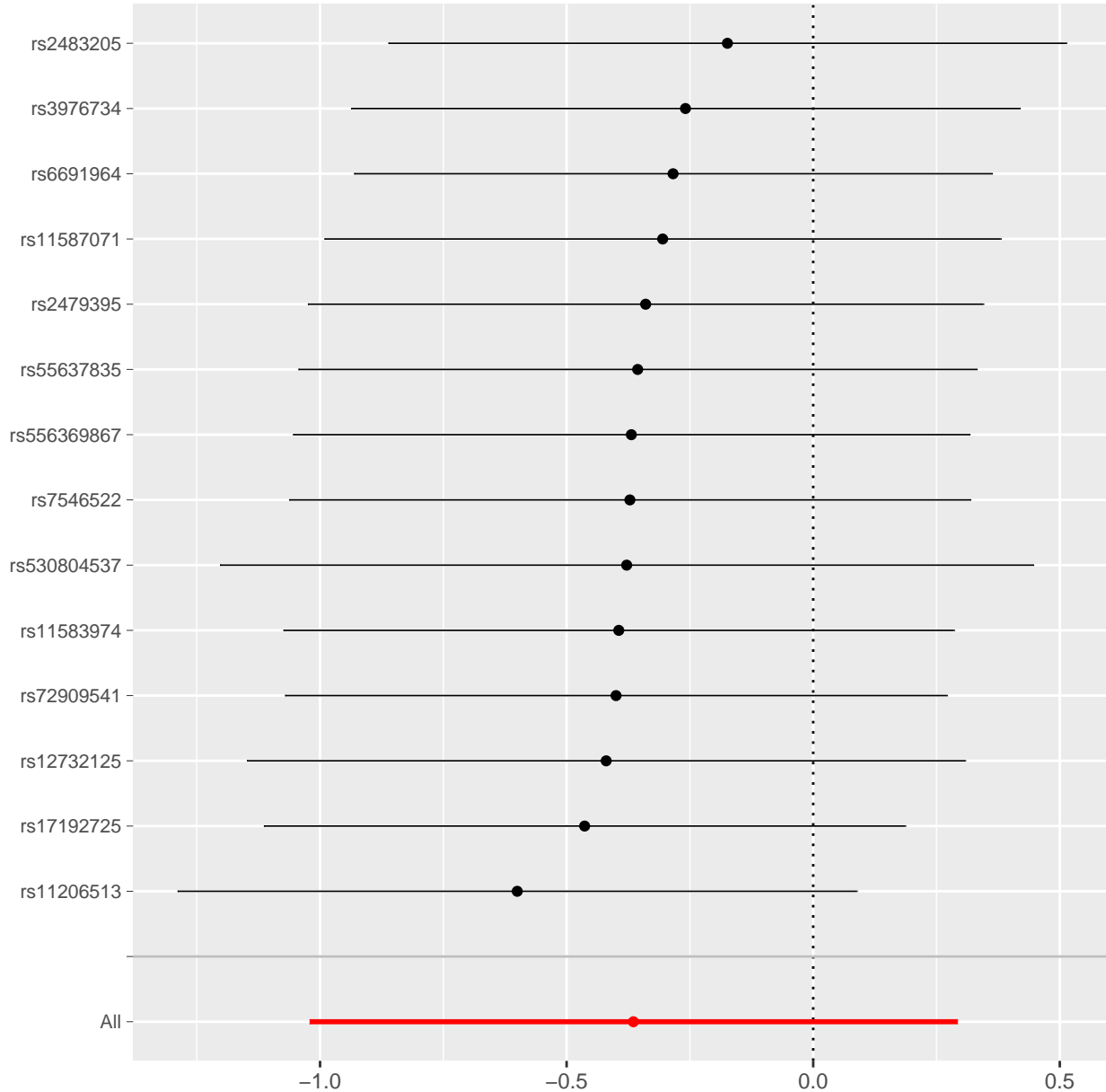

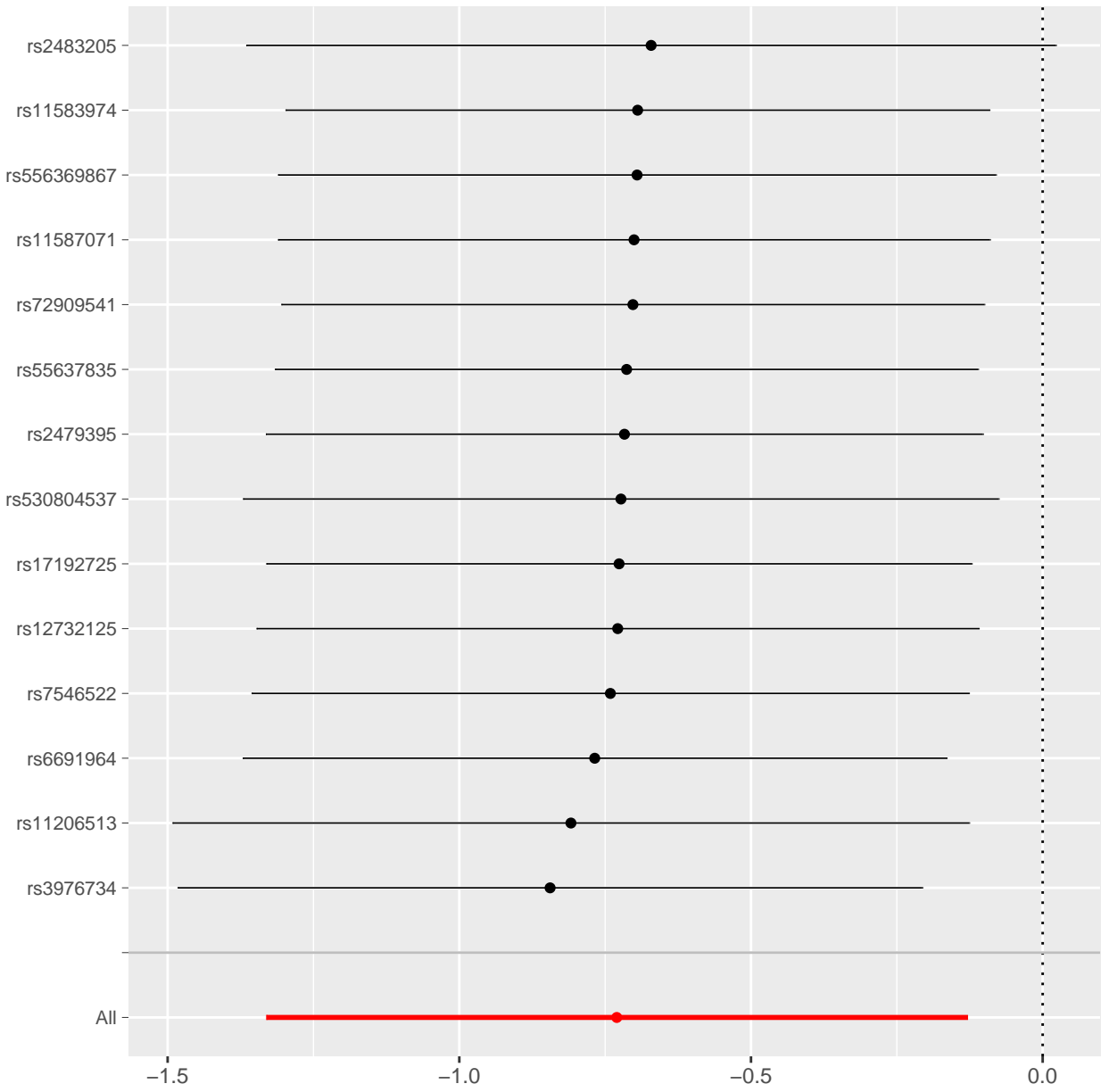

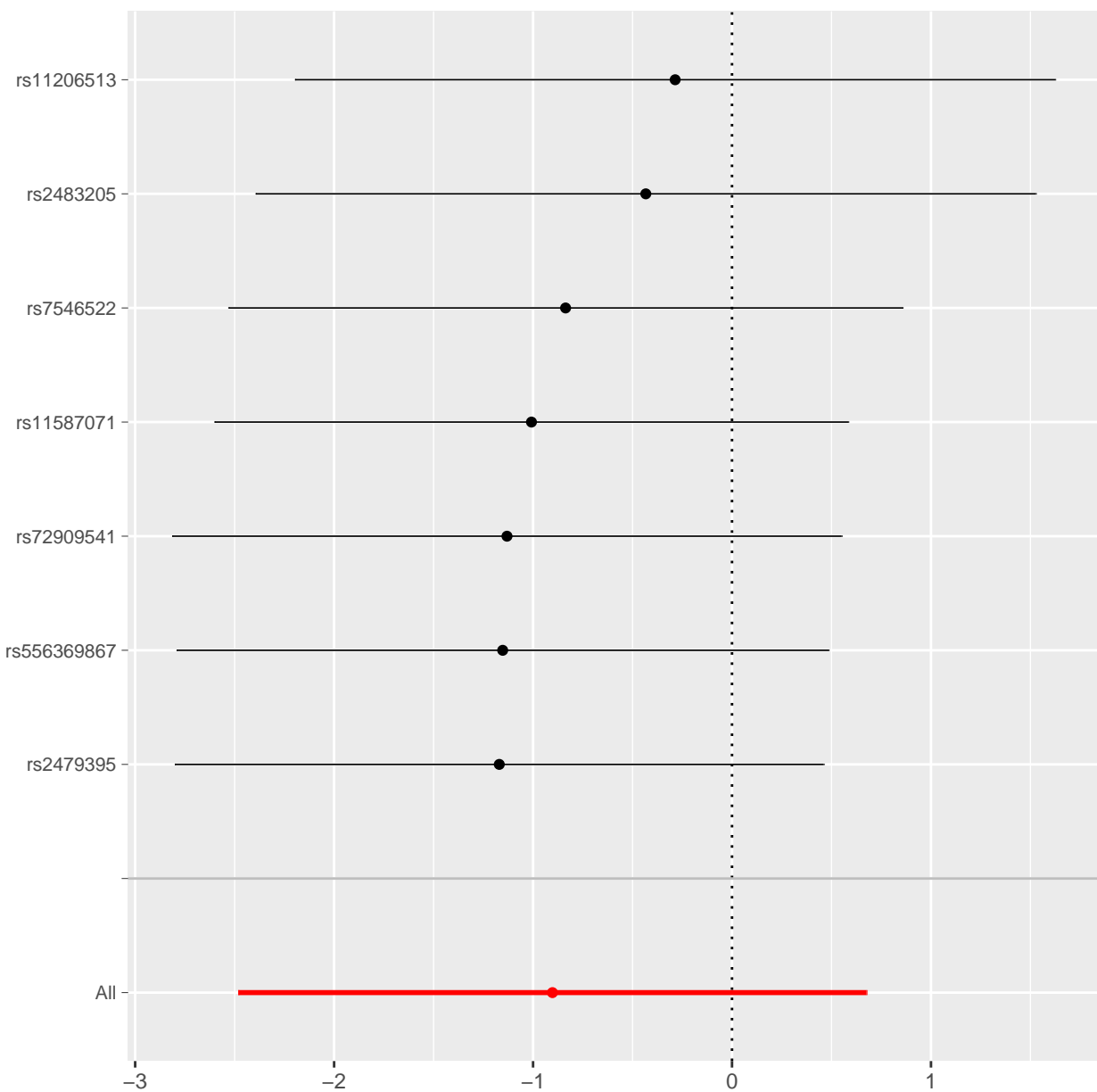

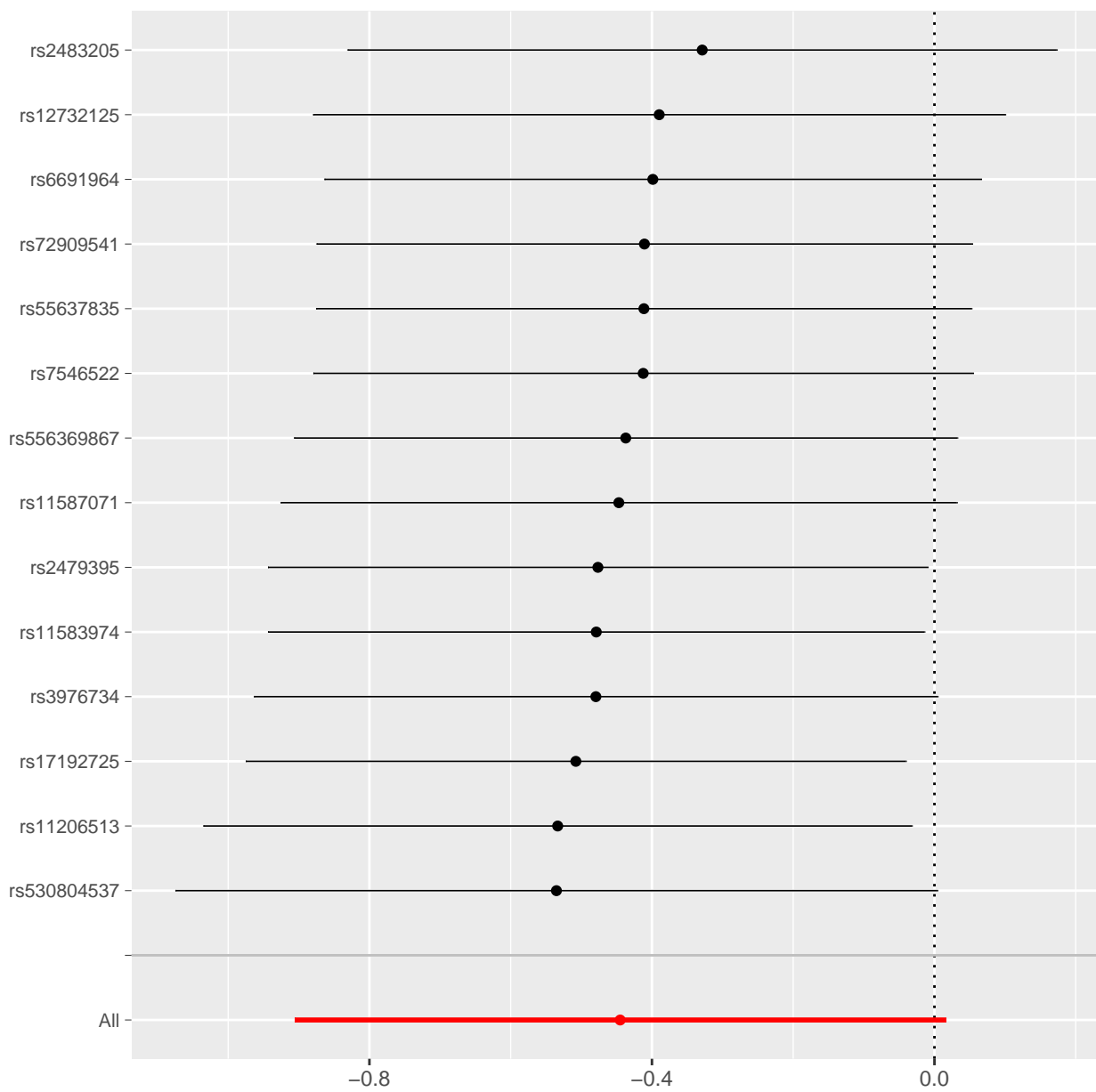

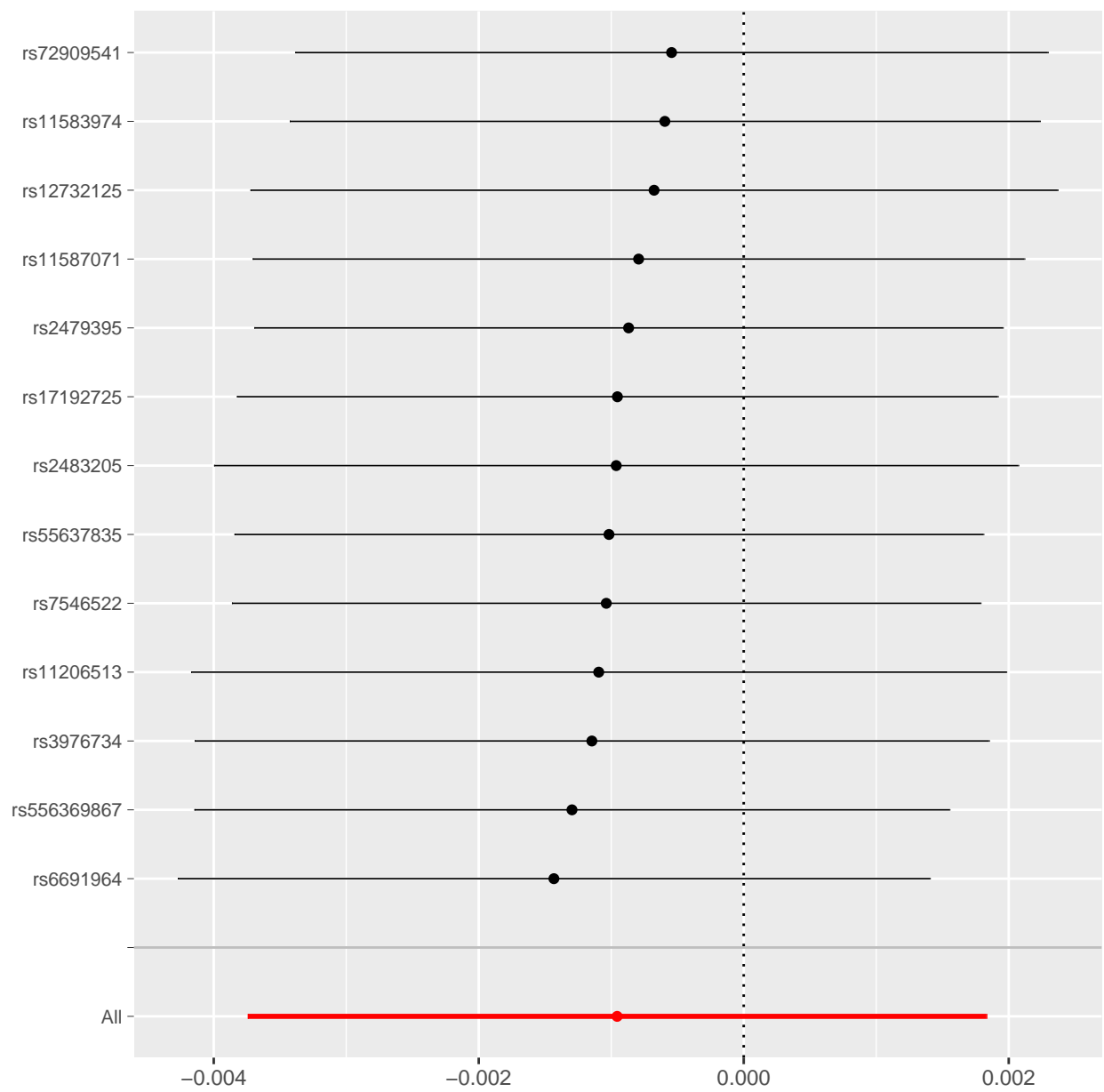

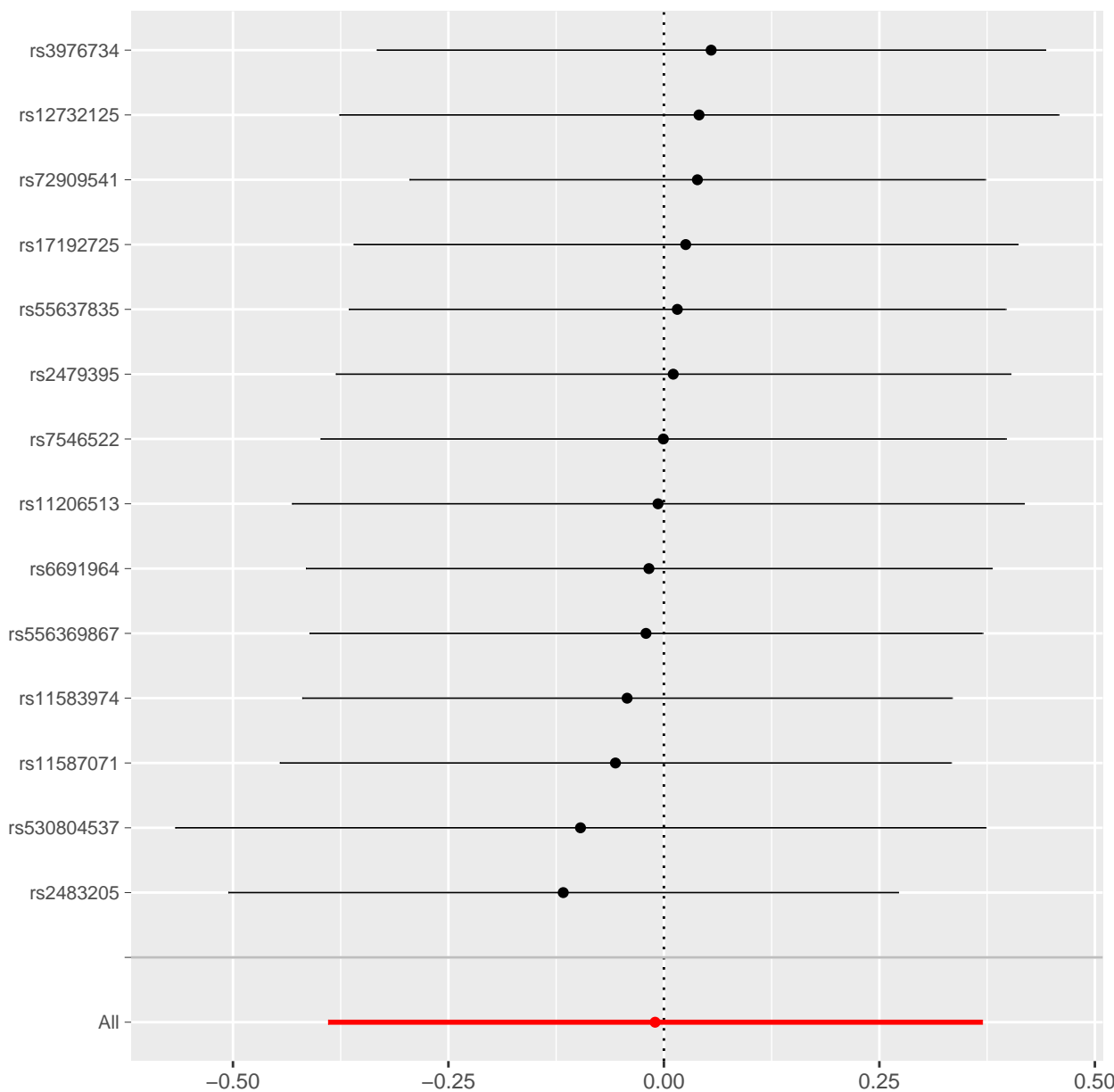

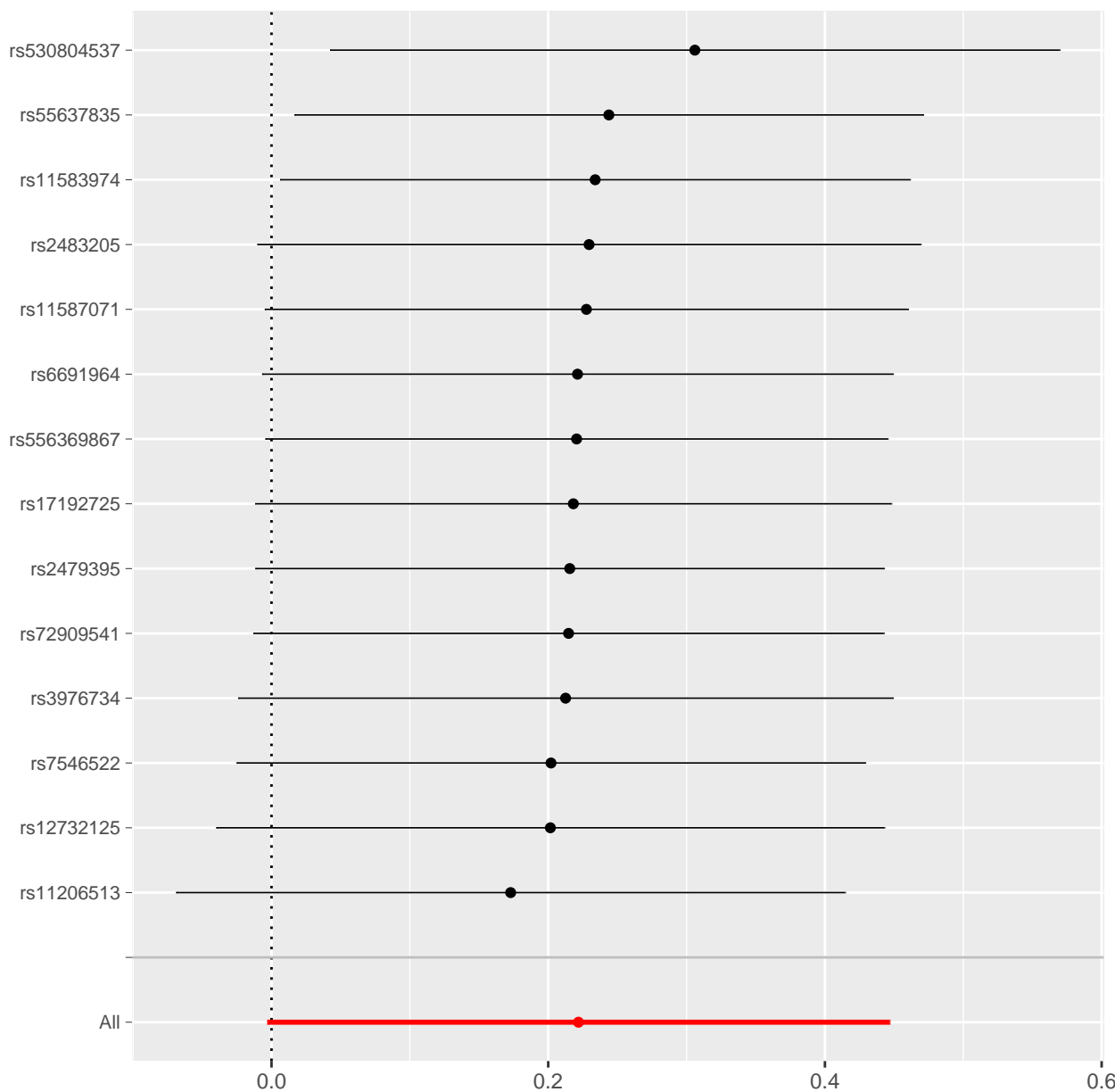

Supplement: Supplementary file 1 [file medi-103-e38010-s001.pdf]

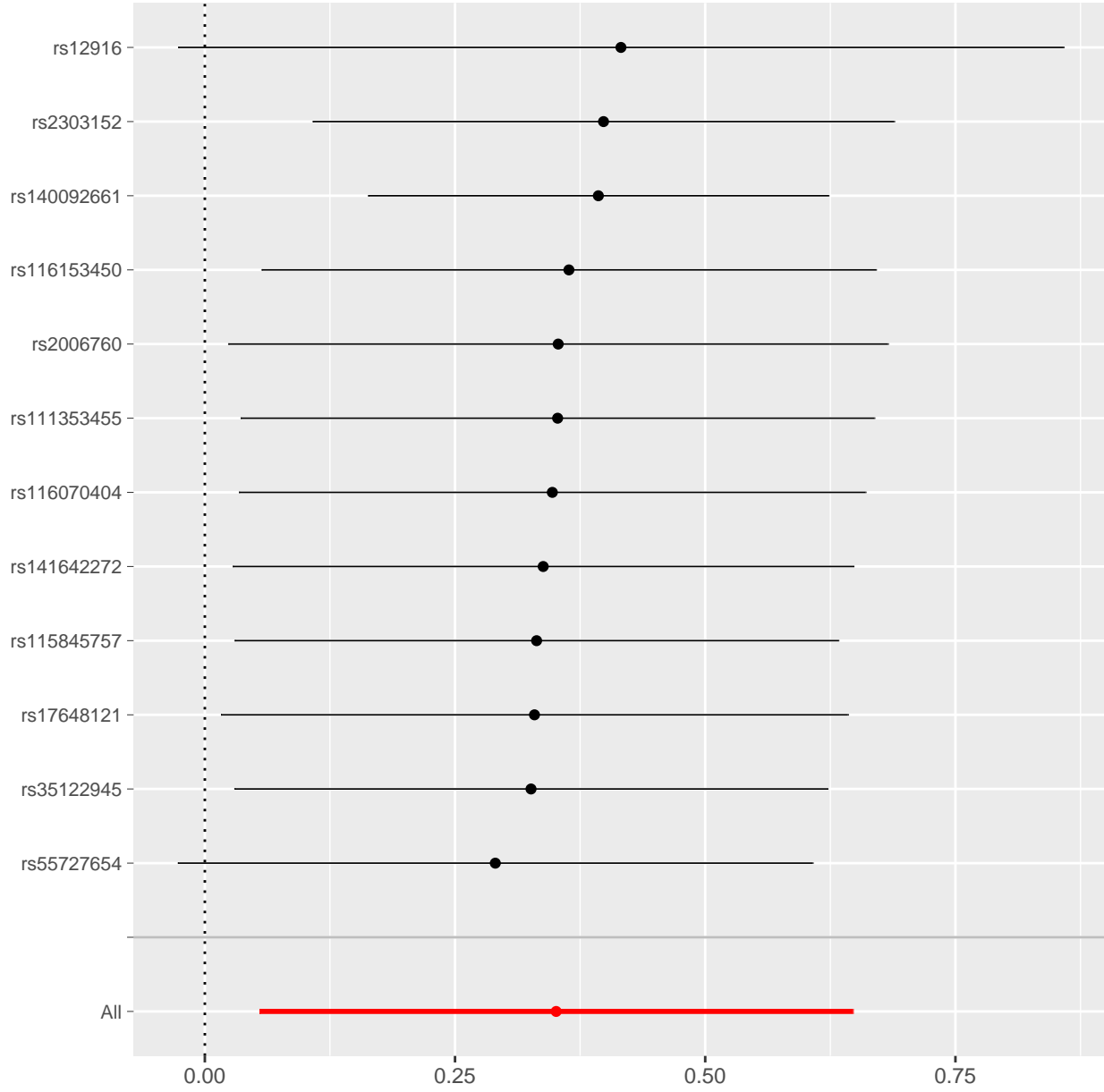

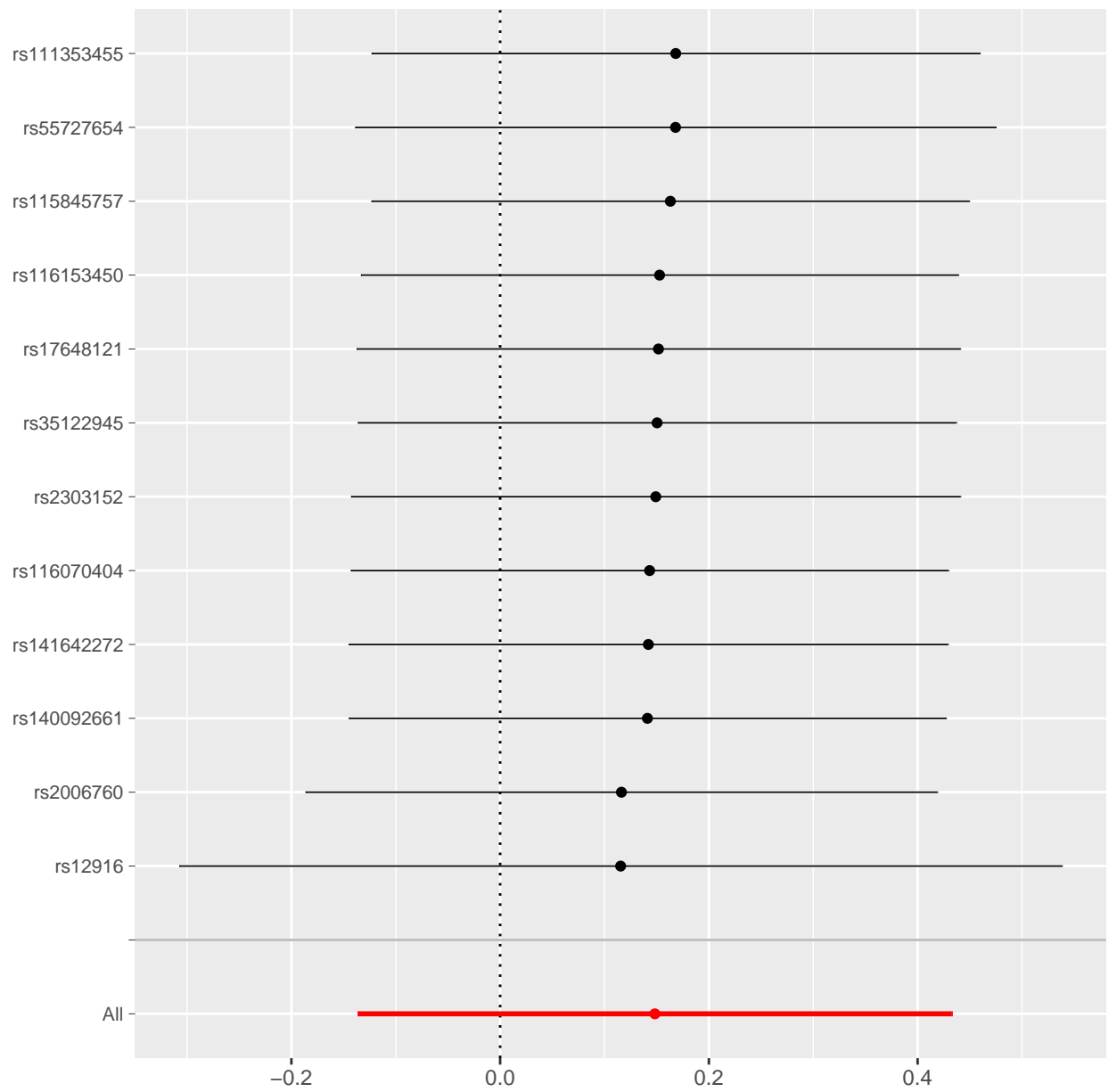

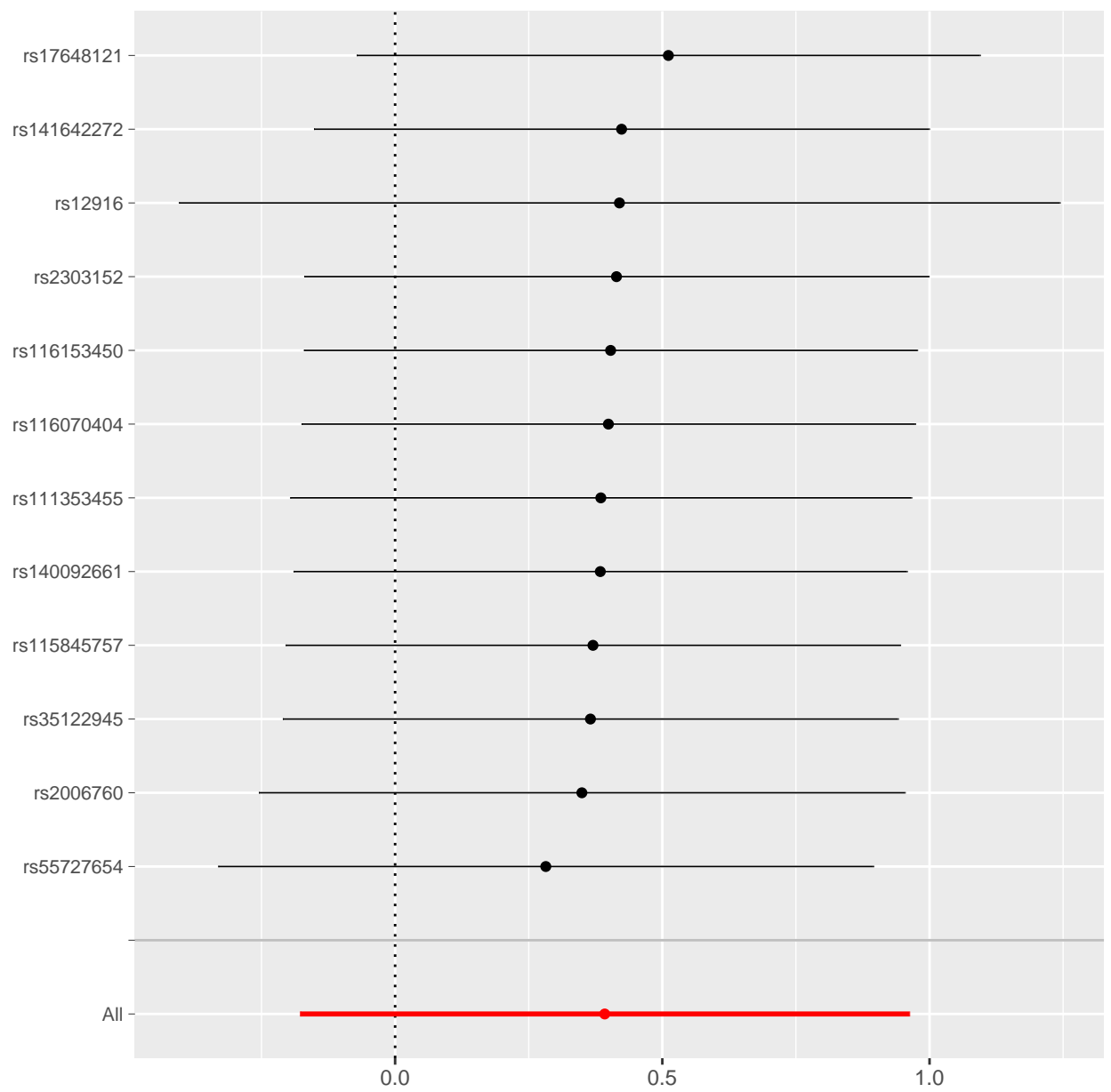

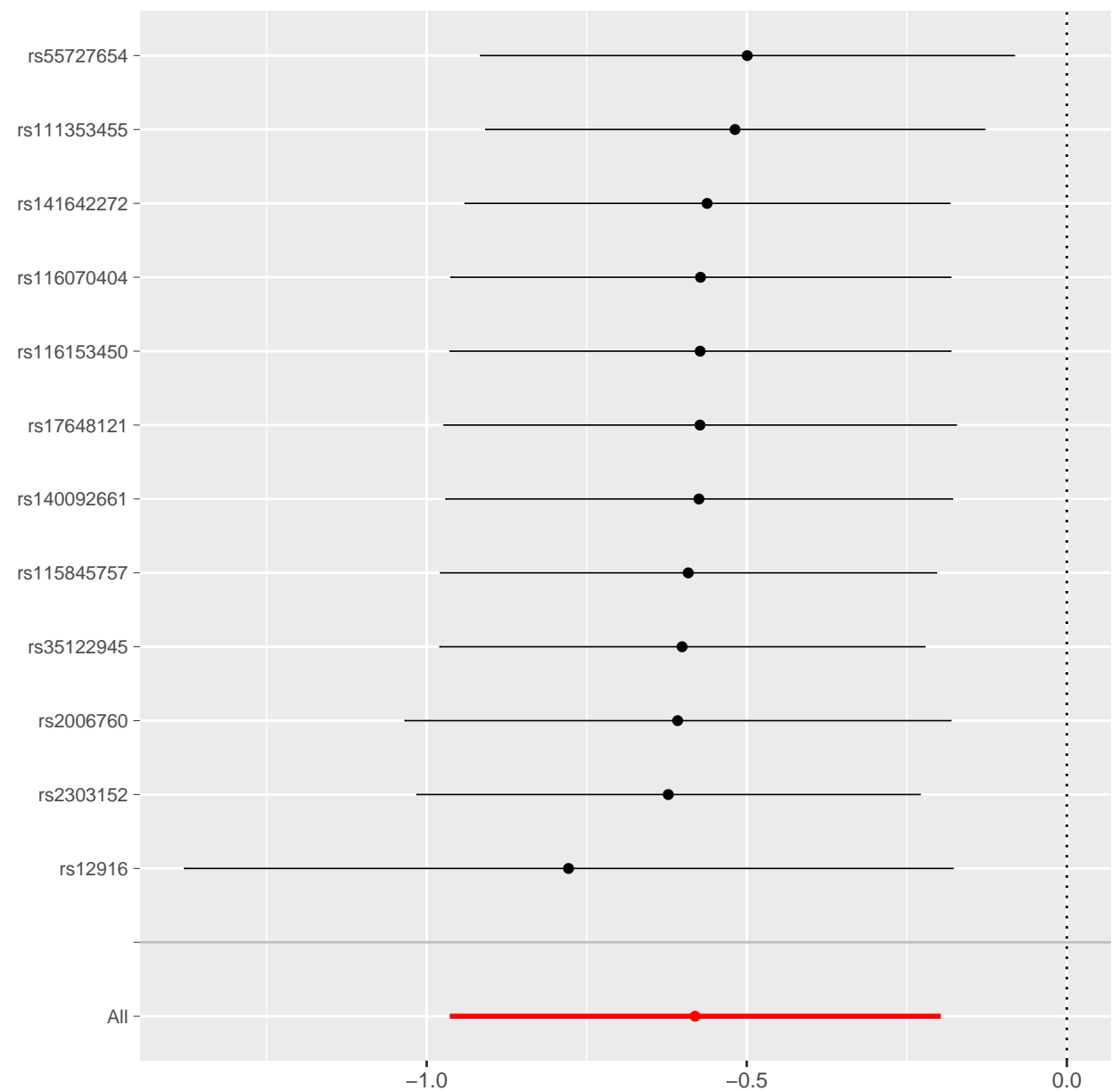

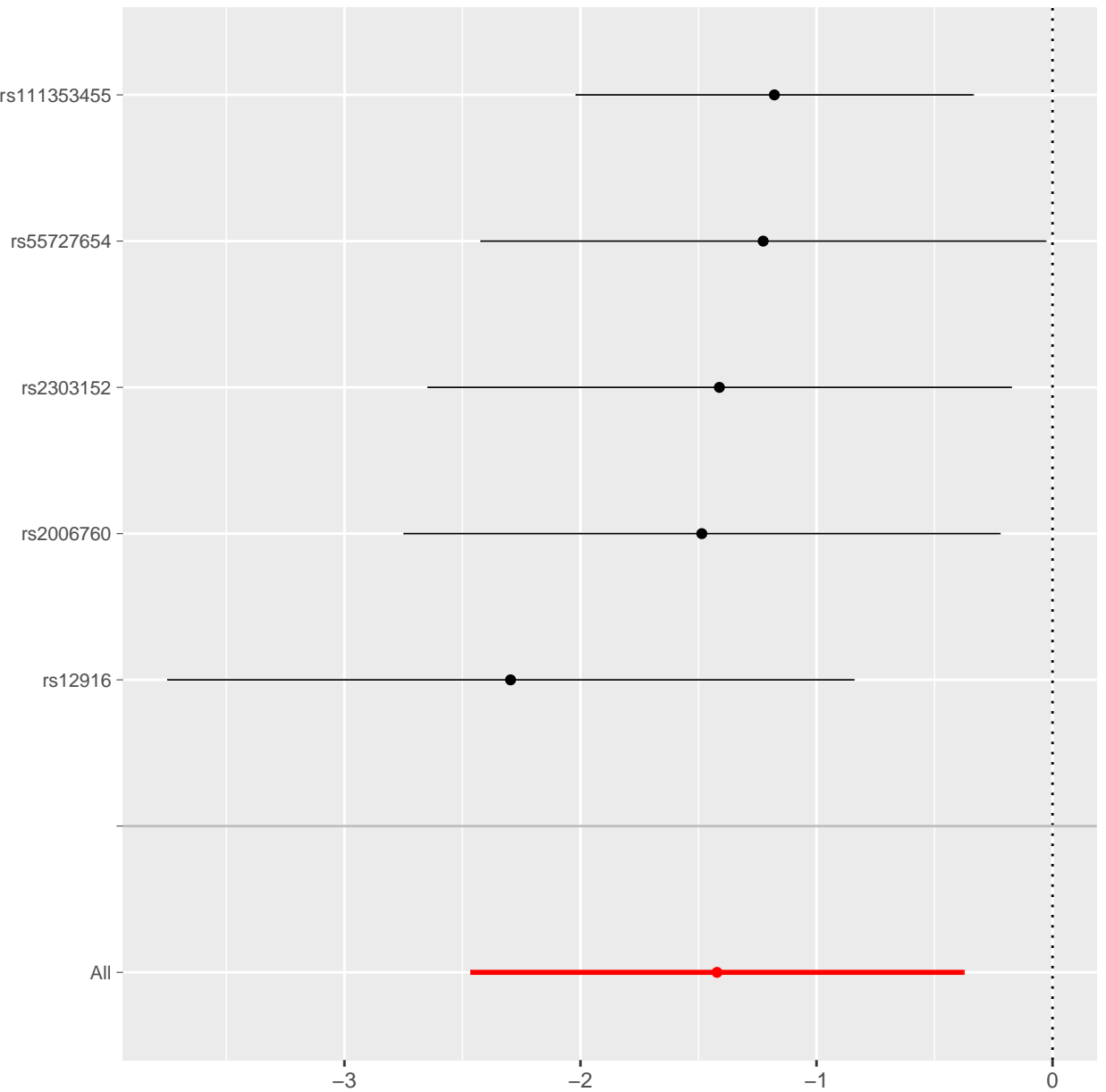

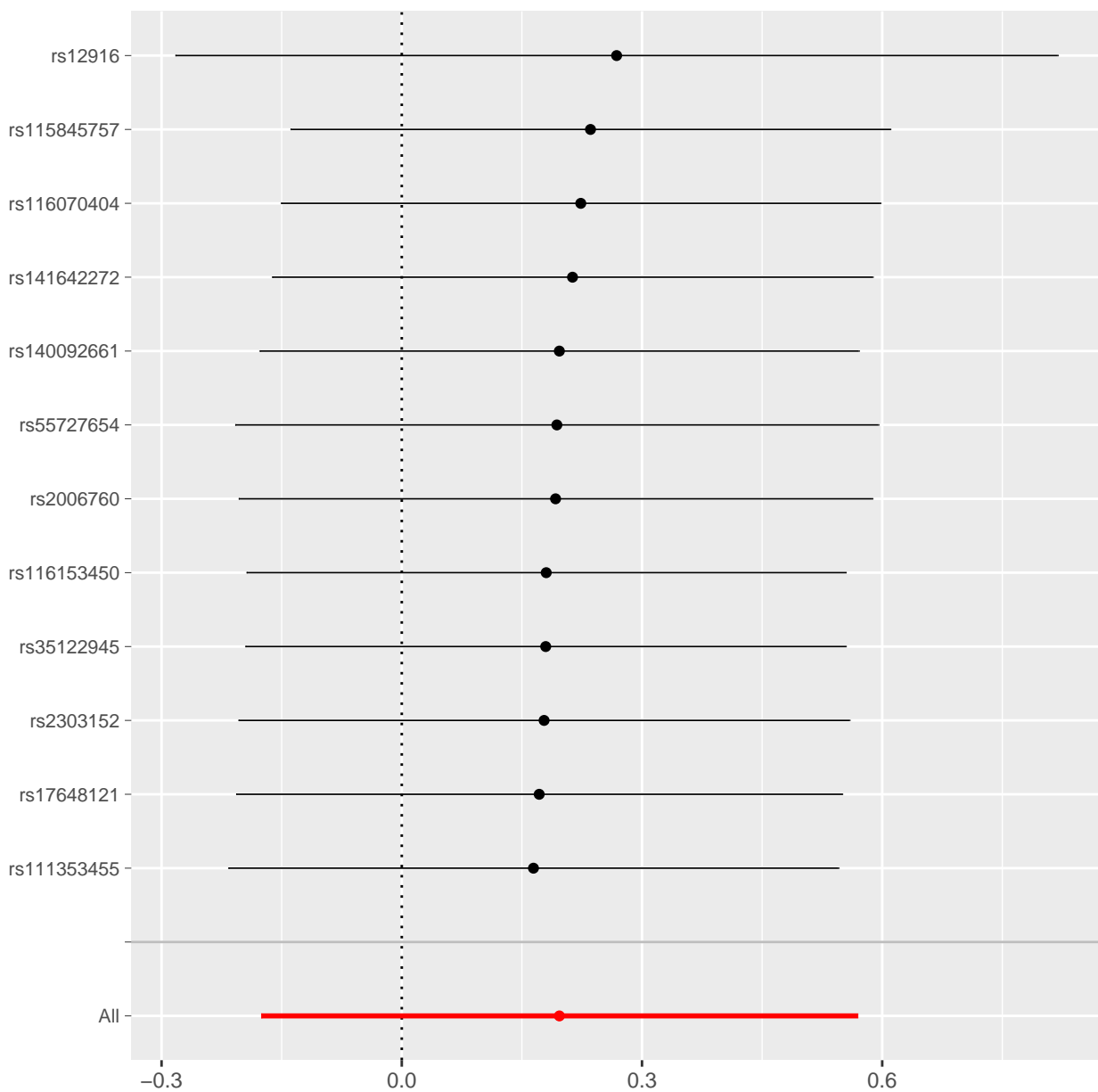

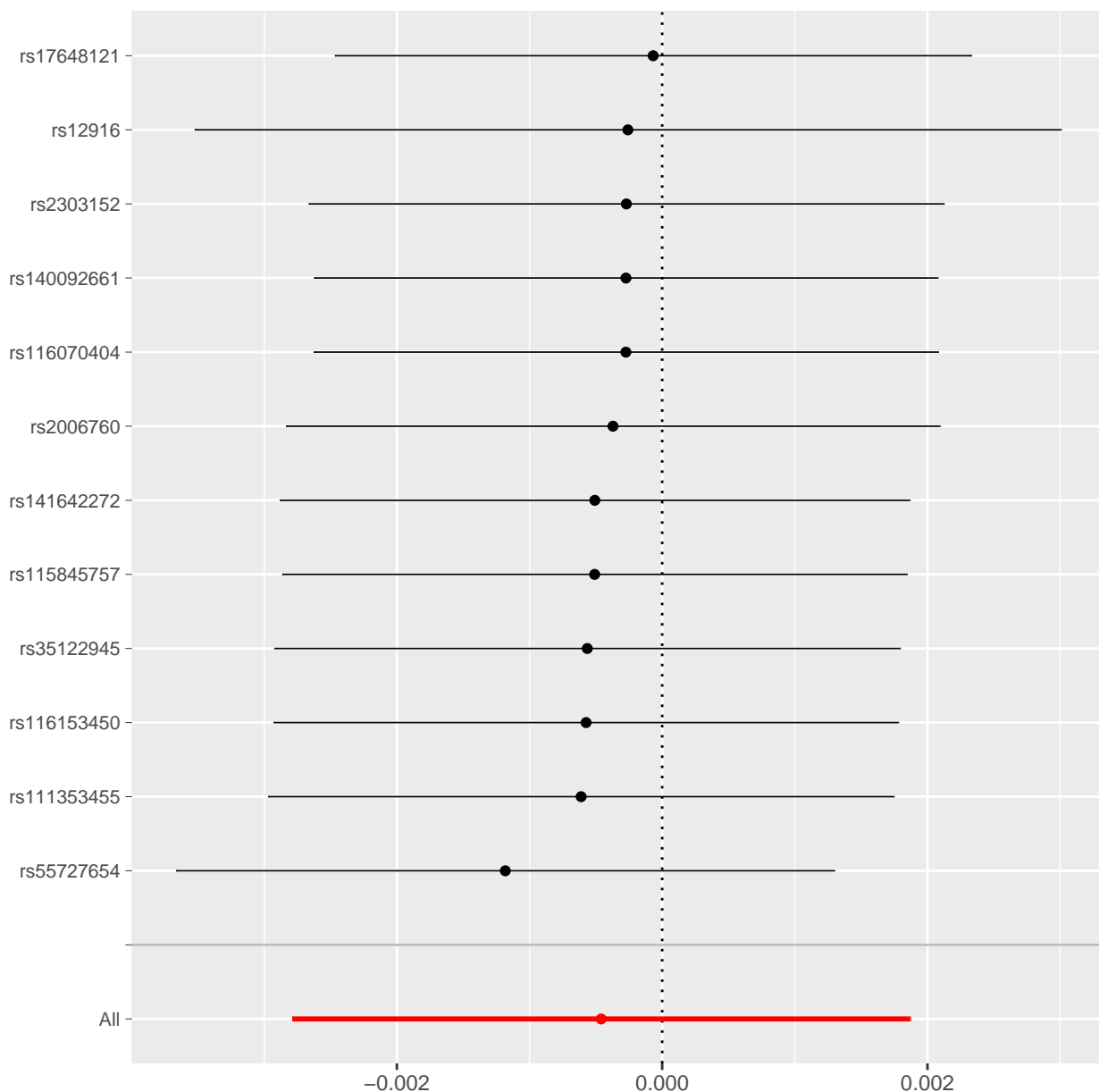

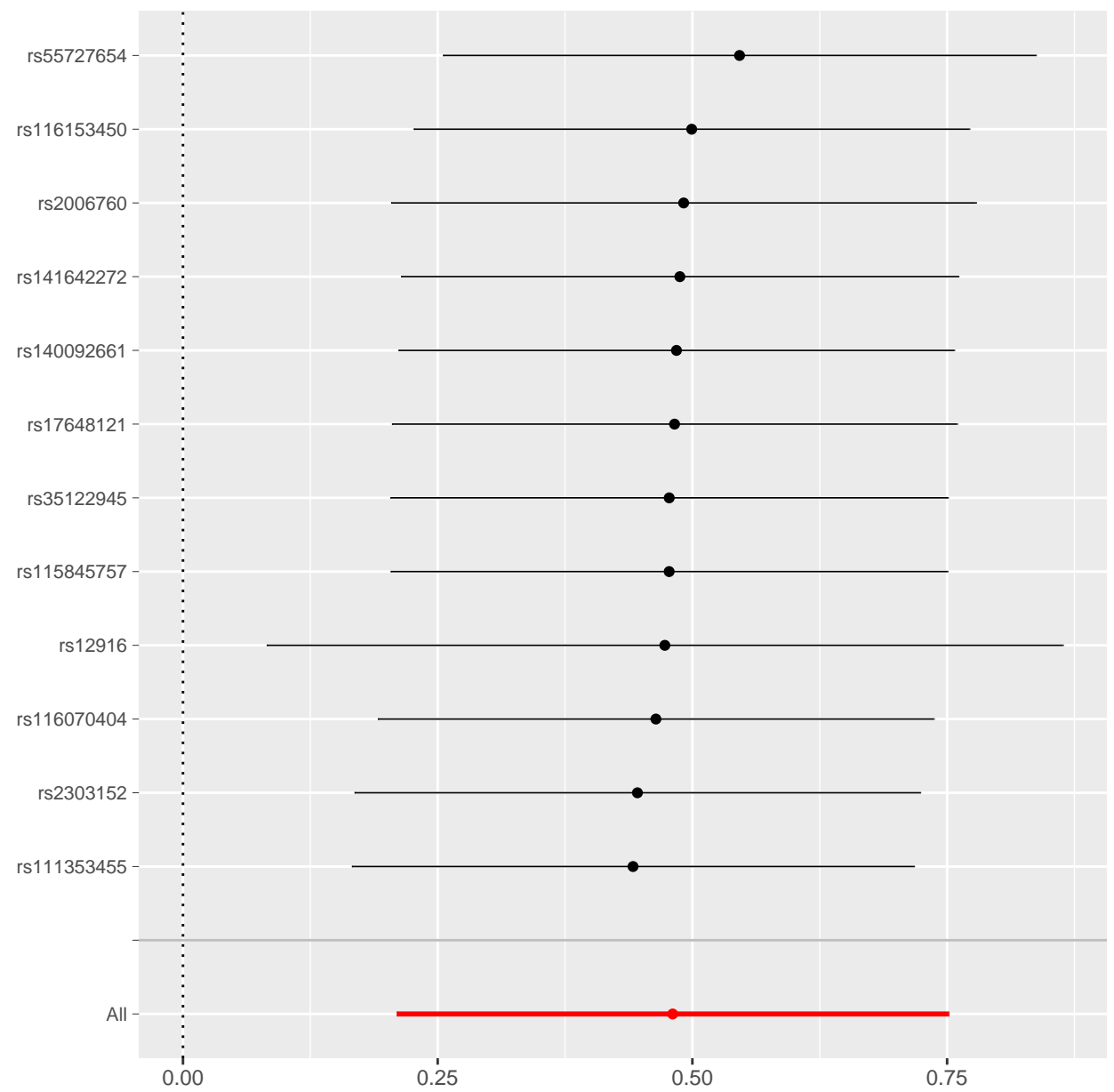

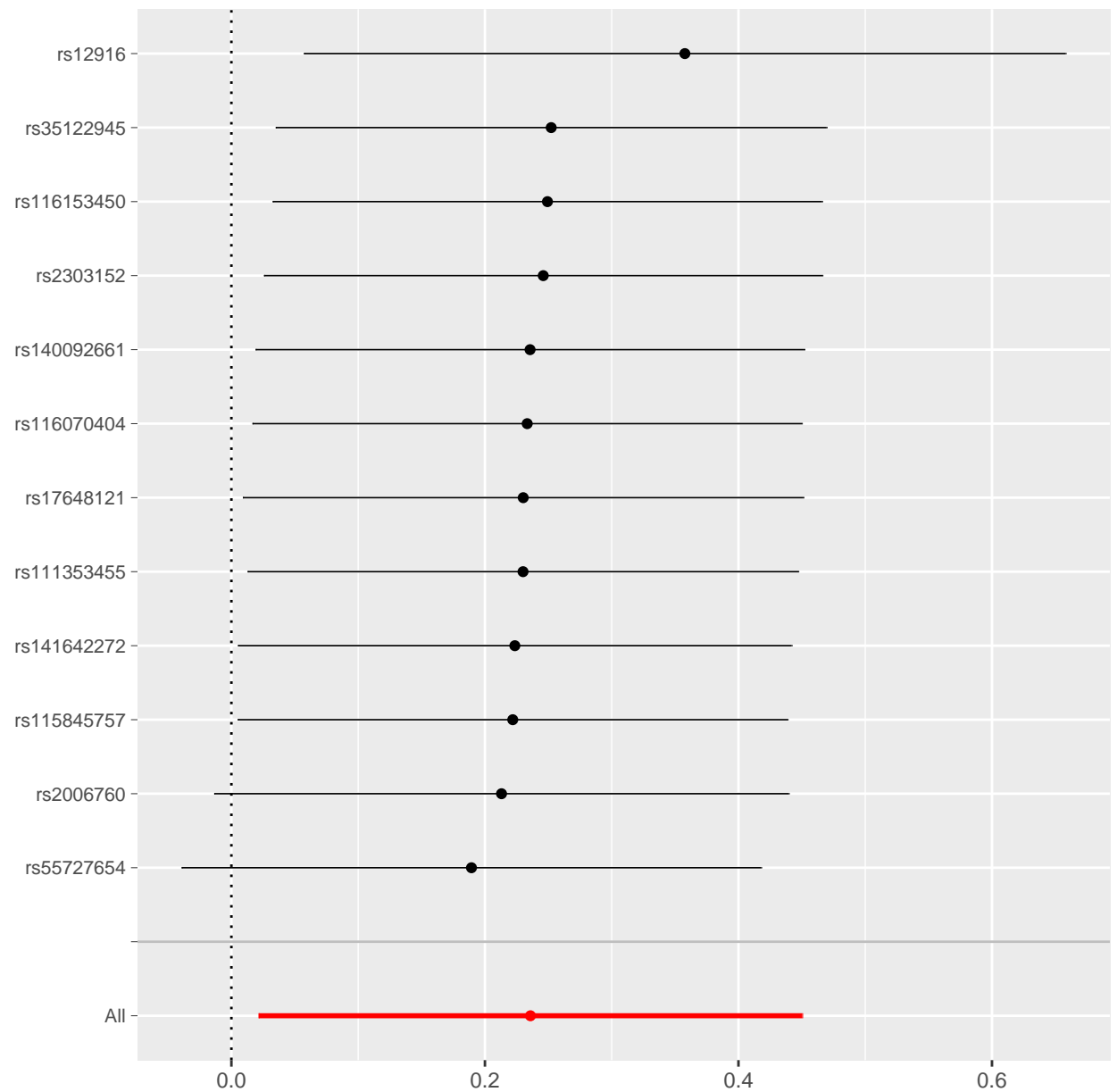

Supplement: Supplementary file 2 [file medi-103-e38010-s002.pdf]
